# A duodenocaval fistula due to erosion of a covered vena cava stent after 10 years

**DOI:** 10.1016/j.jvscit.2025.102081

**Published:** 2025-12-09

**Authors:** Danique J.I. Heuvelings, Diba Demir, Mariëlle M.E. Coolsen, Marc H.A. Bemelmans, Anna Prent, Jorinde H.H. van Laanen

**Affiliations:** a Department of Vascular Surgery, Maastricht University Medical Center+, Maastricht, the Netherlands; b Department of Surgery, Maastricht University Medical Center+, Maastricht, the Netherlands; c Department of General, Visceral and Transplantation Surgery, RWTH Aachen University, Aachen, Germany

**Keywords:** Duodenocaval fistula, Duodenal fistula, Vena cava fistula, Stent erosion, Covered stents, Vena cava inferior pathology

## Abstract

A 58-year-old man developed a rare duodenocaval fistula a decade after cavo-bi-iliac stenting placed for chronic iliocaval occlusion after prior inferior vena cava (IVC) ligation. He presented with fever, polymicrobial bacteremia, and thrombus within the IVC stent. Imaging and endoscopy showed an infected covered stent eroding into the duodenum, creating a fistulous tract. A two-stage surgical approach repaired the damaged duodenum and removed the infected stent, followed by IVC closure using fascia lata. Extensive collateral circulation made reconstruction unsuitable. This case highlights the severe long-term risks of covered IVC stents, particularly in patients with complex venous histories.

A duodenocaval fistula (DCF) is a rare condition. The duodenum's proximity to the inferior vena cava (IVC) increases the chance of a fistula forming only in specific circumstances. In the late 1990s, reports began to emerge describing cases of DCF resulting from peptic ulcers.^1^, ^2^, ^3^ Other reported causes include abdominal injuries such as penetrating trauma or gunshot wounds, perforation of the duodenum by foreign bodies such as toothpicks or bones, and ulceration resulting from radiation therapy.^3^^,^^4^ A 2021 review of enterocaval fistulas (ECFs) identified a total of 52 documented cases of DCF.^5^ The authors reported that approximately 45% of cases were attributed to migrating IVC filters, whereas only two cases were associated with an IVC graft or stent. This report presents an additional case of a 58-year-old man who developed a DCF 10 years after IVC stent placement. Informed consent for publication of this case was obtained from the patient.

## Case presentation

**Fig 1:**
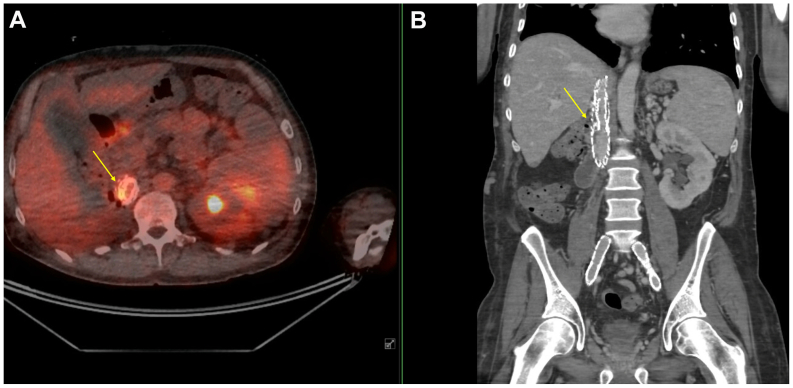
Positron emission tomography-computed tomography (PET-CT) shows hypermetabolism on the stent adjacent to the duodenum. **A,** Transversal PET-CT image. **B,** Coronal CT image. The *yellow arrow* indicates the infected stent.

A 58-year-old man sustained a motor vehicle accident 38 years ago, which necessitated a right-sided nephrectomy. During that procedure, the IVC was ligated because of a bleeding complication. Twenty-eight years after the initial trauma, the patient was re-evaluated after deep venous thrombosis and for symptoms consistent with post-thrombotic syndrome, including complete occlusion of the IVC and involvement of the iliac, femoral, popliteal, and gastrocnemius veins bilaterally. To restore venous outflow, cavo-bi-iliac stenting was performed. This involved the creation of a neo-cava using a covered stent, which was subsequently reinforced with a self-expanding bare-metal venous stent (Sinus XL). The covered endoprosthesis was placed rather than an uncovered venous stent because of an IVC rupture caudal to the liver veins with active bleeding that occurred after balloon angioplasty (Supplementary Video 1, online only). At 1.5 months after the procedure, duplex ultrasound examination revealed approximately 50% stenosis of the reconstructed IVC, with a skip lesion noted between the stents (Fig 1). As a result, a secondary intervention was performed, involving placement of an additional bare-metal stent to optimize flow and reinforce the neo-caval reconstruction (Supplementary Video 2, online only).

**Fig 2:**
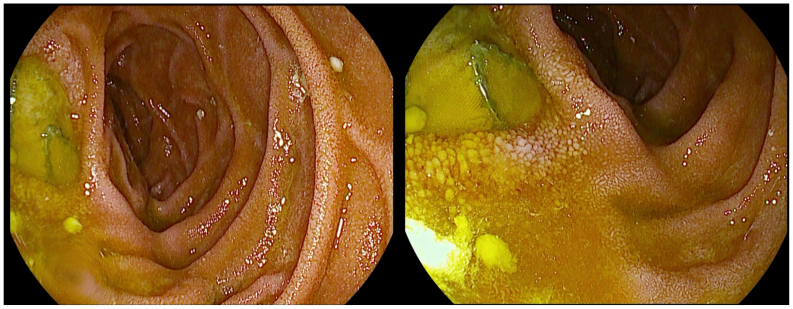
Endoscopic examination of the duodenum shows erosion of the inferior vena cava (IVC) stent extending from the bulb to D2, with an estimated defect of 10 mm.

Thirty-eight years after the initial trauma and 10 years after IVC reconstruction, the patient was admitted to a medical facility abroad with fever and respiratory symptoms. A computed tomography scan revealed findings consistent with pneumonia, along with the presence of a thrombus within the IVC stent and left common iliac stent. Despite receiving both antibiotic and anticoagulant therapy, the patient persisted with high-grade fevers. A subsequent positron emission tomography-computed tomography (PET-CT) scan revealed that the thrombus was infected. Blood cultures identified a polymicrobial infection involving *Escherichia coli*, *Prevotella* species, *Enterococcus faecalis*, and *Candida albicans*, raising suspicion of an ECF. Therefore, he was transported to our academic center. Further workup included a PET-CT, which confirmed the presence of an infected stent adjacent to the duodenum (Fig 1). At the distal end of the covered stent, the horizontal and descending parts of the duodenum lay partially against and behind the IVC stent. Intense hypermetabolism was visible at the contact area between the horizontal part of the duodenum and the stent (Fig 1). Additional endoscopic examination revealed clear erosion of the IVC covered stent into the duodenum, extending from the bulb to the D2 segment, with an estimated defect of approximately 10 mm (Fig 2). No other defects were seen in the further descending part.

Upon admission, the patient was started on targeted antimicrobial therapy in coordination with infectious disease specialists and microbiology support. Given the anticipated complexity of potential reoperation, antimicrobial treatment was maintained for 3 weeks in parallel with prehabilitation measures. Despite this approach, the infection persisted without clinical improvement. After a thorough discussion of management options, the medical team opted for a two-step approach, as performing both procedures at once was deemed too risky (high risk of bleeding during or blowout after stent removal due to stent position just caudal to patent liver veins, compounded by expected adhesions): first, closure of the duodenal defect, followed by removal of the IVC stent only in case additional antibiotic treatment was not sufficient. The procedures were planned and carried out as a collaborative effort between the vascular and hepatopancreatobiliary surgery teams.

Three weeks after admission, surgical closure of the DCF was performed. Extending the initial chevron incision, mobilizing the liver, and lysing adhesions to the small bowel, the duodenum was mobilized using a Kocher maneuver. The duodenum was found to be extensively adherent to the extraluminal covered IVC stent. Careful dissection revealed a large pre-existing duodenal perforation, and a second perforation occurred during the mobilization. Cranial to the covered stent, perforating struts from the Sinus XL bare stent were visible. No active bleeding from the IVC was observed during the procedure. After completing the Kocher maneuver, cholecystectomy was performed to provide access for the placement of a Roder drain into the common bile duct. After the dissection and opening of the duodenum, a splint was introduced into the papilla of Vater, which was located near the edge of the duodenal perforation. It was decided to perform primary closure of both duodenal perforations using polydioxanone sutures Because an iatrogenic pancreatic injury was also identified during the procedure, an additional drain was placed near the site of the injury. A hemostatic agent (TachoSil; Takeda Austria GmbH) was applied over the IVC and the retroperitoneum to support hemostasis and sealing. An omental patch was positioned to reinforce the repair. In addition, a Witzel jejunostomy was created to provide enteral feeding access.

**Fig 3:**
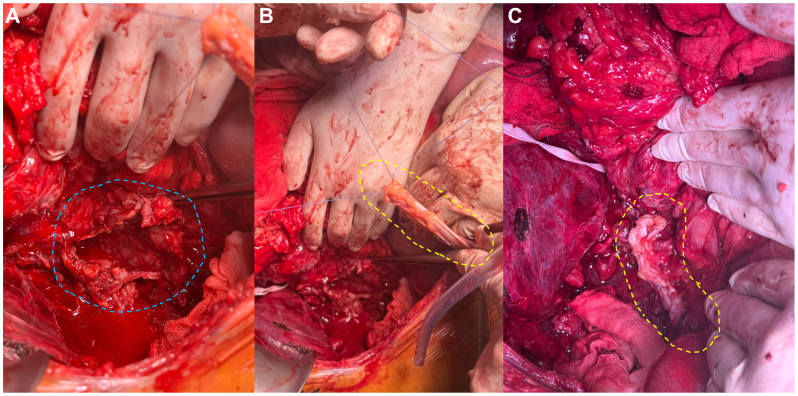
A segment of fascia lata (indicated by the *dotted yellow line*) was harvested from the right upper thigh to be used as a patch on the vena cava. **A,** Defect of the vena cava, indicated by a *dotted blue line*. **B,** Fascia lata graft before implantation. **C,** Fascia lata patch after implantation.

The medical team chose to assess the results of the initial procedure before deciding whether to remove the covered IVC stent. Although the patient ultimately showed clinical improvement, severe adverse effects from extended antibiotic treatment prompted a second intervention 2.5 months after the initial surgery. This follow-up procedure was delayed because of the patient's prolonged recovery from the first operation, largely caused by duodenal and pancreatic leakage. For the second procedure, the same chevron incision was used. Extensive adhesions of the small intestine and colon to the liver and abdominal wall were present. A thorough adhesiolysis was performed, and the liver was mobilized to allow it to be folded medially. The stent was eventually visualized. This process was slow and associated with significant bleeding. The patient became hemodynamically unstable on multiple occasions, necessitating several pauses. Space was carefully created proximally and distally around the stented vena cava to place a clamp. The portal vein was dissected to allow insertion of a perfusion catheter if necessary. A distal clamp was placed just above the iliac bifurcation, and a proximal clamp was placed on the prosthesis. It was decided to transect the IVC distal to the covered section to gain better visualization of a bleeding site caused by distal bleeding from a collateral vessel. The bleeding was controlled, and the distal IVC was repaired with 4.0 Prolene and reinforced with fascia lata. The proximal clamp was moved further proximally, above the covered stent. The Sinus XL bare stent was dissected from the inside. The vena cava wall was additionally dissected proximally, and the stent was carefully maneuvered and removed without bleeding. The large defect in the remnant vena cava (Fig 3, *A*) was closed using 3.0 Prolene and fully reinforced with the fascia lata patch harvested from the right upper thigh (Fig 3, *B* and *C*). The clamp was slowly released, without bleeding of the anastomosis. Extensive hemostasis was performed, and the abdominal cavity was irrigated.

The colon, mobilized laterally from the stent, exhibited serosal damage, necessitating an additional ileocecal resection. Because of hemodynamic instability, an anastomosis was not performed, and a stoma was created instead. The estimated blood loss exceeded 10 L, of which 2.5 L was returned via cell saver, along with transfusion of 10 units of packed cells and 6 units of platelets.

The patient remained in the intensive care unit for 6 days before being transferred to the general ward. One month later, the stoma was successfully revised without complications. Although the overall recovery was prolonged, it was ultimately successful, and the patient was discharged 2 months after the second procedure to continue rehabilitation. During this entire rehabilitation period, antibiotic therapy was maintained. A PET-CT performed approximately 4 months after stent removal demonstrated marked regression of the periprocedural inflammatory area, with only minimal residual fludeoxyglucose uptake, allowing discontinuation of antibiotic treatment. At present, 6 months after stent removal, the patient remains free of any clinical signs of recurrent infection.

## Discussion

This case illustrates a rare occurrence of IVC stent erosion and DCF formation 10 years after the initial endovascular reconstruction aimed at restoring venous outflow. The condition typically presents with intraluminal bleeding, often first identified via endoscopy, though sepsis may also occur.^6^ A prompt surgical intervention is essential to achieve source control and prevent exsanguination, with early surgery linked to improved outcomes.^4^ DCFs carry a mortality risk ranging from 33% to 40%.^4^^,^^5^ Notably, fatal cases of pulmonary embolism caused by the translocation of intestinal contents through a DCF have been reported.^3^ As previously mentioned, only one case in the literature review of Da Cunha et al^5^ was attributed to IVC stent migration, and unfortunately, the patient did not survive because of oncological reasons.^7^ In addition to stenting, that patient also underwent therapeutic radiation after a transabdominal hysterectomy and bilateral salpingo-oophorectomy, which is an added risk factor not present in the patient described in this case.

A more recent case of DCF caused by erosion of an IVC stent without a history of radiotherapy was described by Wang et al.^8^ The case involved a patient who sustained an abdominal gunshot wound, necessitating a duodenojejunostomy and IVC repair. This was later complicated by IVC occlusion, for which a Wallstent was placed. Thirteen years after the initial stent placement, the patient presented with an ECF. This timeframe is comparable to that of the patient presented in the current case; however, a key clinical difference is that this patient experienced abdominal pain in addition to signs of sepsis. Management included partial stent explantation and IVC and duodenal repair, resulting in a patent IVC without signs of reocclusion. The duration of follow-up was not reported by the authors. Our team decided not to perform an IVC reconstruction. Instead, the surgical team opted for definitive IVC ligation, avoiding the high-risk reconstruction involving the stents that remained in situ. The left kidney was not draining into the vena cava anyway, and there was minimal blood flow between the iliac confluence and the liver. Because of extensive collateral formation and longstanding IVC occlusion in our case, the patient was not expected to develop additional complaints of post-thrombotic syndrome.

The management of covered stents in patients with a reconstructed IVC presents a complex clinical challenge. In our case, the decision to initially attempt closure of the duodenal defect, reinforced with an omental patch, while continuing antibiotic therapy, was guided by the anticipated high risk of bleeding and extensive adhesions associated with stent removal. Although simultaneous stent removal and defect closure might have been considered, our approach prioritized patient safety and aimed to stabilize the patient while minimizing perioperative mortality and morbidity. This strategy highlights the need for individualized decision-making in technically complex cases, balancing the potential benefits of definitive intervention against the risks inherent to high-risk vascular and gastrointestinal procedures. Another topic for discussion may be the entire removal of the IVC stents. In this case, the decision was made not to remove the stent immediately. The patient had already undergone two major surgical procedures, and the combination of duodenal/pancreatic leakage and massive hemorrhage might not have been survivable. However, this remains uncertain.

In this case, IVC was likely surgically closed during the trauma surgery 28 years ago. Although venous stenting is generally performed without the use of a covered stent, in this case significant bleeding necessitated placement of a covered stent. Rupture of the iliac vein or IVC with persistent bleeding during endovascular treatment for venous outflow obstruction is extremely rare.^9^ This complication has been reported in a few case studies and can be managed with the placement of a covered or bare-metal stent, along with appropriate resuscitation.^9^, ^10^, ^11^ This case suggests that in patients with a previously ligated IVC, any subsequent recanalization may be entirely extravascular, substantially increasing the risk of severe hemorrhage. It underscores the importance of anticipating the potential need for a covered stent to manage bleeding in similar situations. However, little is known about the tissue response to the covered surface and the associated risk of fistula formation. Accordingly, recanalization in the context of prior IVC ligation should be approached with extreme caution, emphasizing meticulous planning to reduce the risk of life-threatening complications.

## Conclusions

In patients with a history of prior surgery and possible vein ligation, endovenous treatment is likely not the optimal first-line option, as it may increase the risk of hemorrhage, necessitate covered stent placement, and thereby elevate the potential for development of a DCF. This case highlights the importance of informing patients about the small but significant long-term risk of developing a DCF after IVC recanalization. It emphasizes the need for long-term surveillance in patients with IVC stents and supports the feasibility of a multidisciplinary surgical approach for successful management, including duodenal repair and selective stent removal without IVC reconstruction.

## Funding

None.

## Disclosures

None.

## Appendix (online only)

Supplementary Video 1 Cavo-bi-iliac stenting: neo-cava using a covered stent and a self-expanding bare-metal venous stent.

Supplementary Video 2 Cavo-bi-iliac stenting: placement of an additional bare-metal stent to optimize flow.
